# Effects of prebiotic and multispecies probiotic supplementation on the gut microbiota, immune function, and growth performance of weaned piglets

**DOI:** 10.1371/journal.pone.0313475

**Published:** 2024-11-21

**Authors:** Soimer Omar Baldera Huaman, Fernando Augusto de Souza, Melina Aparecida Bonato, Cleandro Pazinato Dias, Marco Aurélio Callegari, Alexandre Oba, Rafael Humberto de Carvalho, Caio Abércio da Silva

**Affiliations:** 1 Department of Animal Science, State University of Londrina, Londrina, Paraná, Brazil; 2 ICC Brazil, São Paulo, Brazil; 3 Akei Animal Research, Fartura, São Paulo, Brazil; University of Life Sciences in Lublin, POLAND

## Abstract

In this study, we evaluated the impact of yeast cell wall prebiotics and multispecies probiotics on the gut microbiota, immune response, and growth performance of weaned piglets, as alternatives to antibiotics as growth promoters (AGPs). A randomized complete block design was employed, involving 160 piglets divided into four treatment groups during the nursery phase. The treatments applied throughout the experimental period were as follows: CONT+ = basal diet with halquinol (AGP); YCW = basal diet with yeast cell wall (cell wall of *Saccharomyces cerevisiae* yeast); SIM+ = basal diet with yeast cell wall + multispecies probiotic (*Bacillus subtilis* (2.0 x 10^9^ CFU/g), *Bacillus coagulans* (5.0 x 10^8^ CFU/g), *Clostridium butyricum* (5.0 x 10^7^ CFU/g), and *Bacillus licheniformis* (2.0 x 10^9^ CFU/g)); SIM- = basal diet with yeast cell wall + multispecies probiotic (half dose). The parameters assessed included daily feed intake, weight gain, feed conversion ratio (FCR), diarrhea score, serum cytokine levels, and chemokine concentrations, as well as microbiota analysis. During the 21 to 63-day study period, only FCR differed significantly (p = 0.0076). CONT+ and PREB had superior FCRs of 1.543 and 1.585, while SIM- had the least favorable FCR at 1.654. At 35 days, IL-10 levels were greater in the SIM- group, showing a 271.25% increase over those in the other groups. By 49 days, the IL-8 concentration was lower in the PREB group than in the CONT+ group, with a reduction of 247%, while the IL-8 concentrations in the SIM+ and SIM- groups were not significantly different from those in the other groups. The *Firmicutes*/*Bacteroidetes* (F/B) ratio in the CONT+ group was lower than that in the PREB, SIM+, and SIM- treatment groups. The *Lactobacillaceae* family was more abundant in the SIM+ treatment, followed by the SIM- and PREB treatments. The CONT+ treatment had the lowest abundance. The abundance of the genus *Lactobacillus* differed between the CONT+ group and the PREB, SIM+, and SIM- treatment groups. Prebiotics, used either alone or combined with probiotics, serve as effective substitutes for AGPs, boosting piglets’ health and performance throughout the nursery phase.

## Introduction

The weaning of piglets at commercial farms is normally carried out between 21 and 28 days of age, a process that marks a critical period, especially within the initial two weeks following the separation from sows [1]. Nevertheless, potential adverse effects may persist throughout the nursery phase, impacting piglet development [2].

A primary consequence of postweaning stress is a reduction in feed intake. Depending on the severity and duration, this reduction can impair the functionality of the intestinal barrier. This impairment is characterized by morphological changes in the intestine, including alterations in villus height and crypt depth [3]. Such intestinal morphophysiological modifications can disrupt the activity of digestive enzymes, leading to osmotic diarrhea. This condition, coupled with diminished nutrient absorption, facilitates the proliferation of *Escherichia coli*. Bacteria exploit available substrates for growth, thereby exacerbating digestive disturbances [4]. After weaning, an increase in feed consumption is positively correlated with modulation of the intestinal microbiota, enhancing organ health. This relationship is critical for promoting the proliferation of beneficial bacteria, especially by enriching lactobacillus populations and depleting *Clostridium coccoides* in the ileum [5].

Therefore, antibiotics as growth promoters (AGPs) have historically been used to mitigate postweaning challenges. However, recent regulations have curtailed their use due to concerns over the emergence of antibiotic-resistant bacteria and potential residues in meat [6]. Consequently, this shift has favored the adoption of alternatives such as prebiotics and probiotics, whose beneficial effects on animal health are increasingly recognized [7–10]. These developments underscore the importance of nutritional strategies in supporting the intestinal health and overall well-being of weaned piglets, reflecting a move toward more sustainable and health-conscious farming practices.

Probiotics confer various benefits to the host’s intestinal health through multiple mechanisms, such as the competitive exclusion of pathogens, the production of antimicrobial substances, the of enterotoxins, modulation of the host’s immune response, and preservation of intestinal barrier integrity [11,12]. Similarly, prebiotics enhance gut health by preventing bacterial adhesion to the intestinal wall, modulating immunity via antibacterial compounds, promoting secretion within the intestinal lumen, and inducing beneficial morphological changes in the intestinal structure [13].

Probiotics, prebiotics, and their combination (symbiotic) are recognized for their ability to modulate the intestinal microbiota, thereby enhancing intestinal health and fostering the development of both the local and systemic immune systems. This modulation influences piglet performance [10,14–19]. Given the multifaceted nature of weaning-related stress and the diverse range of available probiotics and prebiotics with varying compositions, concentrations, dosages, and active ingredients, this study aimed to assess the efficacy of a prebiotic, both alone and in combination with a probiotic, in comparison to that of an AGP. This study addresses the need for effective alternatives to antibiotic growth promoters in swine production by evaluating the combination of prebiotics and probiotics. Our research focuses on sustainable farming practices that prioritize animal health while reducing the risks associated with AGP use. The focus was on evaluating their effects on the intestinal microbiota and immune system and their subsequent impacts on the health and performance of piglets during the nursery phase.

## Materials and methods

All procedures conducted in this study were thoroughly reviewed and approved by the AKEI Animal Research Ethics Committee for Animal Experimentation under approval number 007/22. The experiment involved 160 piglets, evenly divided between castrated males and females, of PIC^®^ genetics (Camborough x AG 337), which were aged 21 days and had an average live weight of 5.5 ± 1.0 kg. The experimental design was a randomized complete block (based on initial animal weight and sex) comprising four treatments with eight replicates each, and the experimental unit consisted of five animals of the same sex. Throughout the experimental period (21 to 63 days of age), the animals had *ad libitum* access to water and feed, and the nutritional program (Table 1) was divided into four phases: prestarter I (21 to 28 days), prestarter II (29 to 35 days), starter I (36 to 42 days), and starter II (43 to 63 days).

**Table 1:** Composition and nutritional values of the diets used in the treatments according to the feeding phases.

Ingredients (%)	Phases			
	Prestarter I	Prestarter II	Starter I	Starter II
Corn (7.5%)	15.434	29.873	38.612	59.725
Soybean meal (45.0%)	20.710	22.462	29.011	31.930
Extruded corn	35.000	25.000	15.000	0.000
Milk whey	16.000	12.000	8.000	0.000
Powdered milk	6.000	4.500	2.000	0.000
Soy protein concentrate	2.000	2.000	1.000	0.000
Soybean oil	0.664	0.000	2.393	3.892
Dicalcium phosphate	1.179	1.202	1.421	1.841
Limestone	0.088	0.531	0.539	0.709
HCl lysine (80%)	0.718	0.540	0.446	0.427
L-Threonine (98%)	0.360	0.230	0.207	0.157
DL-Methionine (98%)	0.318	0.262	0.203	0.131
L-Tryptophan (98%)	0.067	0.066	0.056	0.047
L-Valine	0.056	0.164	0.017	0.026
Copper sulfate	0.064	0.062	0.061	0.049
Choline chloride 60%	0.053	0.004	0.000	0.000
Salt	0.477	0.474	0.543	0.577
Zinc oxide 72% Zn	0.401	0.220	0.082	0.081
Butylhydroxytoluene 99%	0.010	0.010	0.010	0.010
Mycotoxin adsorbent	0.150	0.150	0.150	0.150
Vitamin premix ^1^	0.150	0.150	0.150	0.150
Mineral premix ^2^	0.100	0.100	0.100	0.100
**Total**	100.00	100.00	100.00	100.00
**Nutrients**				
Metabolizable energy, Kcal/kg	3450	3400	3400	3400
Crude protein, %	18.50	19.00	19.80	19.50
Crude fat, %	5.05	3.37	5.47	6.44
Crude fiber, %	2.65	2.85	3.19	3.47
Calcium, %	0.55	0.69	0.70	0.80
Phosphorus available, %	0.45	0.40	0.42	0.42
Digestible lysine, %	1.44	1.33	1.30	1.24
Digestible meth + cys, %	0.85	0.81	0.78	0.70
Digestible threonine, %	0.94	0.85	0.85	0.79
Digestible tryptophan, %	0.25	0.24	0.25	0.23
Digestible valine, %	0.75	0.89	0.82	0.84
Sodium, %	0.35	0.30	0.30	0.25
Chlorine, %	0.82	0.70	0.61	0.47

^1^Vitamin premix (levels per kg of product) included the following: vitamin A: 6,000 IU; vitamin D3: 1,500 IU; vitamin E: 15,000 mg; vitamin K3: 1,500 mg; vitamin B1: 1,350 mg; vitamin B2: 4,000 mg; vitamin B6: 2,000 mg; vitamin B12: 20 mg; niacin: 20,000 mg; pantothenic acid: 9,350 mg; folic acid: 600 mg; and biotin: 80 mg.

^2^Mineral premix (levels per kg of product) included the following: iron: 100 mg; copper: 10 mg; manganese: 40 g; cobalt: 1,000 mg; zinc: 100 mg; iodine: 1,500 mg; and selenium: 300 mg.

The treatments applied throughout the experimental period were as follows: CONT+ = basal diet with 200 g/ton halquinol (AGP); PREB = basal diet with 1000 g/ton prebiotic; SIM+ = basal diet with 500 g/ton prebiotic + 600 g/ton multispecies probiotic; SIM- = basal diet with 500 g/ton prebiotic + 300 g/ton multispecies probiotic.

The prebiotic derived from the cell wall of *Saccharomyces cerevisiae* yeast (ImmunoWall^®^, ICC, Brazil) consisted of 1,3–1,6 β-glucans (>35%) and mannan oligosaccharides (>19%). The multispecies probiotic used (Bio4Pro^®^, PHARTEC SAC, Lima, Peru) contained a blend of spore-forming strains, including *Bacillus subtilis* (2.0 x 10^9^ CFU/g), *Bacillus coagulans* (5.0 x 10^8^ CFU/g), *Clostridium butyricum* (5.0 x 10^7^ CFU/g), and *Bacillus licheniformis* (2.0 x 10^9^ CFU/g).

In this study, we evaluated daily feed intake (DFI), daily weight gain (DWG), and the feed conversion ratio (FCR), and the results are presented for each phase and for the entire experimental period (21 to 63 days). Diarrhea scores were recorded daily and classified as follows: normal consistency (0), soft (1), pasty (2), and watery (3) [20]. The average diarrhea severity score was calculated using the following formula:


Averagediarrheaseverityscore=totaldiarrheascore/totalnumberofanimalsevaluatedovertheexperimentalperiod.

At 35 and 49 days of age, the levels of serum cytokines and chemokines (IL-1β, IL-4, IL-6, IL-8, IL-10, IL-12p40, IFN-α, IFN-γ, and TNF-α) were quantified. Blood samples (5 mL) were collected via jugular vein puncture from one animal per pen (32 animals in total), centrifuged, and then analyzed for a panel of 9 cytokines using a multiplex immunoassay designed for pigs (Invitrogen™, EPX090-60829-901). At 35 days of age, fecal samples were collected from the rectum, for one sample per experimental unit, totaling 32 samples, for microbiota analysis. This evaluation was conducted using a ZR Fecal DNA MiniPrep^®^ kit from Zymo Research (Murphy Ave., Irvine, CA) following the manufacturer’s recommended protocol for DNA extraction. The extracted DNA was quantified using spectrophotometry at 260 nm, and its integrity was assessed through electrophoresis on a 1% agarose gel. A segment of approximately 460 bases from the hypervariable V3-V4 region of the 16S rRNA ribosomal gene was amplified using universal primers under the following PCR conditions: 95°C for 3 minutes; 25 cycles of 95°C for 30 seconds, 55°C for 30 seconds, and 72°C for 30 seconds; and a final step at 72°C for 5 minutes. The resulting amplicons were used to construct a metagenomic library using a Nextera DNA Library Preparation Kit from Illumina^®^. The amplicons were pooled and sequenced on an Illumina^®^ MiSeq sequencer [21].

Animals that were deemed unfit for treatment and recovery due to health or injury reasons, as determined by a veterinarian, and were therefore unable to remain in the study, were euthanized. Euthanasia was performed by first desensitizing the animals using the electronarcosis procedure (with a minimum current of 1.3A for 3 seconds and a minimum voltage of 240V), followed by the severing of the vessels in the neck region.

The sequencing reads were analyzed on the QIIME2 platform (Quantitative Insights Into Microbial Ecology) [22], following a workflow to remove low-quality sequences, filter reads, remove chimeras, a perform taxonomic classification. Sequences were classified as bacterial genera on the basis of amplicon sequence variants (ASVs) determined based on homology compared to a database. This analysis was performed with the 2021 version (GTDB release 202) of the Genomic Taxonomy Database for ribosomal sequences [23]. To classify bacterial communities by identifying ASVs, a total of 16,633 reads per sample was used to normalize the data and prevent comparison of samples with varying read numbers.

The normality of the distribution of the data was analyzed using the Kolmogorov‒Smirnov & Lilliefors test and the Shapiro‒Wilk W test (p>0.05). The Box & Whisker package was used to remove outliers. Normally distributed data were subjected to analysis of variance (ANOVA) using a general linear model (GLM), with the model considering block and treatment effects. The means from this analysis were further evaluated using Tukey’s test. Both analyses were performed using Statistics for Windows^®^ software, version 10.0 [24]. For the test results, a p-value equal to or less than 0.05 was considered to indicate significance, and a p-value between 0.05 and 0.10 was considered to indicate a trend.

Immunity parameters were analyzed using GraphPad Prism version 8; Gaussian-distributed data were subjected to one-way ANOVA with Tukey’s multiple comparisons post-test, while non-Gaussian data were subjected to the Kruskal‒Wallis test, followed by Dunn’s multiple comparisons post-test. For the fecal microbiota analyses, statistical comparisons of alpha diversity among each analyzed group were conducted using the nonparametric Kruskal‒Wallis test and Dunn’s post hoc test, with results deemed statistically significant at p-values less than 0.05. Beta diversity statistical analysis was performed using Permutational Multivariate Analysis of Variance in the QIIME2 pipeline, with 10,000 permutations. Alpha diversities were calculated using "phyloseq" [25], "vegan" [26], and the "microbiome" library [27]. Differences in the relative abundance of taxa between groups were estimated using the Kruskal‒Wallis test and Dunn’s post hoc test.

## Results

During the prestarter I (21 to 28 days of age) and prestarter II (29 to 35 days of age) phases, there were no differences in zootechnical performance among treatment groups (Table 2; p>0.05). In the starter I phase (36 to 42 days of age), the CONT+ group (0.276 g) showed greater (p = 0.012) DWG than did the SIM+ (0.214 g) and SIM- (0.237 g) treatment groups, while the PREB treatment group (0.245 g) did not differ from the other groups, presenting an intermediate value. In the starter I phase, the feed conversion ratio (FCR) was lowest in the CONT+ group (1.600), indicating better efficiency (p = 0.020). Higher FCRs, reflecting less efficient feed conversion, were observed in the SIM+ (1.985), PREB (1.795), and SIM- (1.773) groups, with no significant differences between these treatment groups. In the starter II phase (43 to 63 days of age), the CONT+ group (1.532) tended to have a greater FCR (p = 0.096) than the SIM- group (1.648), with no differences found for the PREB and SIM+ groups compared to the other treatment groups. No differences were detected for the other parameters in this phase among treatments.

**Table 2:** Mean Live Weight (LW), Daily Weight Gain (DWG), Daily Feed Intake (DFI) and Feed Conversion Ratio (FCR) of piglets in the weaning phases provided diets containing prebiotics and multispecies probiotics.

Parameters	Treatments				CV (%)	p-value
	CONT+	PREB	SIM +	SIM -		
	Prestarter I (21–28 d)					
LW21d (kg)	5.555	5.757	5.500	5.521	16.96	0.952
DFI (kg)	0.129	0.125	0.132	0.137	17.57	0.848
DWG (kg)	0.082	0.068	0.070	0.070	42.78	0.926
FCR	1.669	1.958	2.060	2.125	29.71	0.740
	Prestarter II (29–35 d)					
LW29d (kg)	5.761	6.104	5.801	5.905	14.42	0.868
DFI (kg)	0.285	0.309	0.300	0.294	14.80	0.754
DWG (kg)	0.216	0.233	0.223	0.207	21.34	0.740
FCR	1.326	1.347	1.377	1.452	14.11	0.597
	Starter I (36–42 d)					
LW36 (kg)	7.277	7.740	7.363	7.356	13.53	0.818
DFI (kg)	0.435	0.438	0.417	0.416	12.02	0.761
DWG (kg)	0.276a	0.245ab	0.214b	0.237b	14.22	0.012
FCR	1.600a	1.795ab	1.985b	1.773ab	12.60	0.020
	Starter II (42–63 d)					
LW42 (kg)	9.213	9.457	8.862	9.016	11.84	0.738
DFI (kg)	0.838	0.845	0.800	0.812	9.19	0.607
DWG (kg)	0.546	0.539	0.505	0.495	9.65	0.144
FCR	1.532a	1.568ab	1.589ab	1.648b	5.63	0.097
LW63 (kg)	20.685	20.796	19.472	19.415	9.83	0.362
	Total (21–63 d)					
DFI (kg)	0.556	0.567	0.539	0.545	9.20	0.731
DWG (kg)	0.360	0.358	0.332	0.330	9.23	0.150
FCR	1.543a	1.585ab	1.625bc	1.654c	3.79	0.008

^a, b, c^ Different letters in the rows indicate a significant difference as determined by the Tukey test (p<0.05) or trend (p<0.10). CONT+ (200 g halquinol/ton of feed); PREB (1,000 g prebiotic/ton of feed); SIM+ (500 g prebiotic + 600 g probiotic/ton of feed); SIM- (500 g prebiotic + 300 g probiotic/ton of feed).

Throughout the entire experimental period, from 21 to 63 days of age, a difference was observed only in the FCR (p = 0.0076). The CONT+ (1.543) and PREB (1.585) groups exhibited the best results, with no significant difference between them, while the SIM- (1.654) treatment group had the poorest outcome.

Regarding the number of animals with diarrhea and the diarrhea index (Table 3) throughout the experimental period, differences were observed for score 2 (p = 0.010), where animals in the CONT+ (n = 19) and SIM+ (n = 18) groups exhibited 35.71% and 28.57% more diarrhea, respectively, compared to those in the SIM- (n = 14) group, with the PREB (n = 16) treatment group being intermediate, showing 14.28% more diarrhea. For score 3, there were 46.66% more piglets in the PREB group (n = 22) than in the SIM- treatment group (n = 15), while 13.33% and 6.66% more piglets in the CONT+ (n = 17) and SIM+ (n = 16) groups experienced diarrhea, respectively, than in the SIM- group (n = 15). For scores 2 and 3 combined, there was a greater percentage of animals in the CONT+ (n = 36) and PREB (n = 38) groups (p = 0.010) than in the SIM- (n = 29) treatment group (24.13% and 31.06%, respectively), with the SIM+ (n = 34) group showing a 17.24% greater incidence.

**Table 3:** Number of piglets with diarrhea and diarrhea indices according to experimental treatments.

Score	Treatments				p-value
	CONT+	PREB	SIM +	SIM -	
Score 1 (n)	1	0	0	0	0.4015
Score 2 (n)	19a	16ab	18a	14b	0.0100
Score 3 (n)	17ab	22a	16ab	15b	0.0100
Score 2 + 3 (n)	36a	38a	34ab	29b	0.0100
Diarrhea index	0.018	0.019	0.017	0.015	-
Observations (n)	2000	1971	1985	1949	-

^a ‐ b^ Different letters in the rows indicate differences according to the GLM with Tukey’s post hoc test (p≤0.05). CONT+ (200 g halquinol/ton of feed); PREB (1,000 g prebiotic/ton of feed); SIM+ (500 g prebiotic + 600 g probiotic/ton of feed); SIM- (500 g prebiotic + 300 g probiotic/ton of feed).

For the majority of the 9 serum cytokines quantified at the two ages (Table 4), no difference was detected among treatments, with the exceptions being the level of IL-10 (at 35 days of age), which was 271.25% greater in the SIM- group than in the other treatment groups, and the level of IL-8 (at 49 days of age), which was lower (-247%) in the PREB group than in the CONT+ group, while the SIMB+ and SIMB- groups did not differ from the others.

**Table 4:** Mean values of the concentrations of pro- and anti-inflammatory cytokines in piglets fed the experimental treatments at 35 and 49 days of age.

	Treatments						C.V. (%)		p-value	
	CONT+	PREB		SIM+		SIM-				
35 d of age										
IL-1β (pg/mL)	6.960	3.846		16.35		17.86	73.83		0.0799	
IL-4 (pg/mL)	1.787	0.9883		2.194		2.243	64.58		0.1498	
IL-6 (pg/mL)	3.775	3.737		3.768		2.899	99.33		0.3666	
IL-8 (pg/mL)	2.515	2.515		2.597		2.515	216.44		0.3430	
IL-10 (pg/mL)	2.832b	2.832b		2.832b		7.682a	119.78		0.0125	
IL-12p40 (pg/mL)	415.8	273.4		273.3		361.3	84.60		0.9466	
IFN-α (pg/mL)	0.9524	0.6063		0.8284		1.124	209.24		0.7359	
IFN-ϒ (pg/mL)	2.368	2.368		2.368		2.368	0.00		1.0000	
TNF-α (pg/mL)	3.381	3.381		3.381		3.381	0.00		1.0000	
49 d of age										
IL-1β (pg/mL)	7.876	4.532	7.013		5.303			146.65		0.4916
IL-4 (pg/mL)	1.029	1.152	0.9748		1.029			79.03		0.9337
IL-6 (pg/mL)	3.488	3.784	3.775		3.748			29.03		0.5398
IL-8 (pg/mL)	4.624a	1.872b	2.058ab		2.515ab			53.94		0.0261
IL-10 (pg/mL)	2.832	2.832	9.862		9.918			109.42		0.0952
IL-12p40 (pg/mL)	488.1	300.1	257.7		283.5			64.80		0.4882
IFN-α (pg/mL)	0.5153	0.5780	1.353		0.4276			86.36		0.2098
IFN-ϒ (pg/mL)	2.368	2.368	2.368		2.368			0.00		1.0000
TNF-α (pg/mL)	3.381	3.381	3.381		3.381			0.00		1.0000

^a ‐ b^ Different letters in the rows indicate a difference according to the Kruskal‒Wallis test (p<0.05), with Dunn’s post hoc test. CONT+ (200 g halquinol/ton of feed); PREB (1,000 g prebiotic/ton of feed); SIM+ (500 g prebiotic + 600 g probiotic/ton of feed); SIM- (500 g prebiotic + 300 g probiotic/ton of feed). C.V.: coefficient of variation.

**Fig 1:**
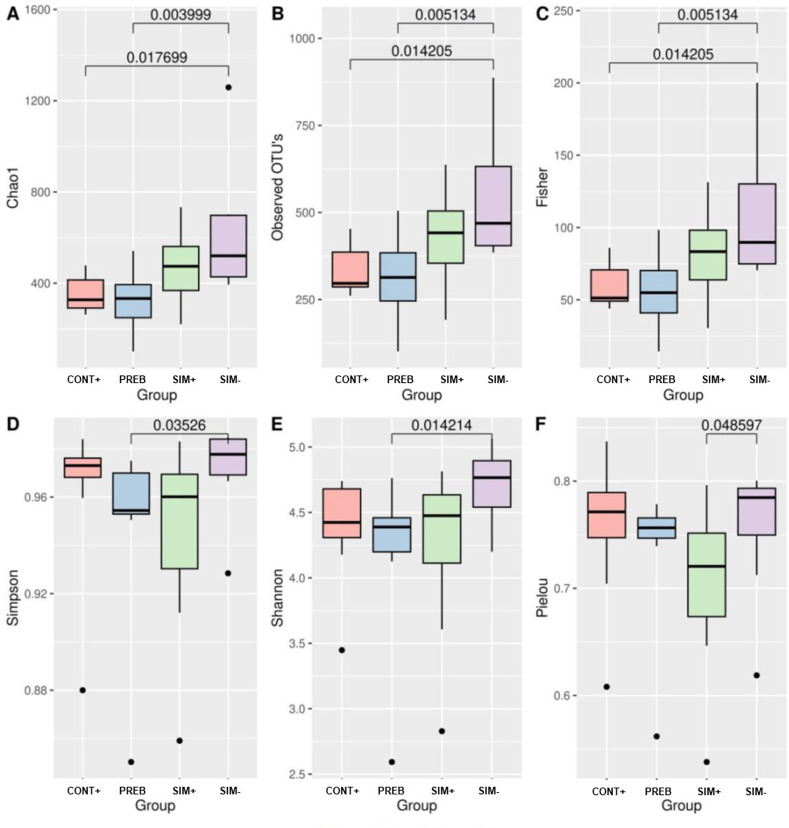
Alpha Diversity Estimated by the Parameters Chao 1 (A), Observed OTUs (B), Fisher (C), Simpson Index (D), Shannon Entropy (E), and Pielou’s Evenness (F). Statistical comparisons between results with different treatments were conducted using the nonparametric Kruskal‒Wallis test and Dunn’s post hoc test. The differences in results with statistical values less than 0.05 were considered significant. CONT+ (200 g halquinol/ton of feed); PREB (1,000 g prebiotic/ton of feed); SIM+ (500 g prebiotic + 600 g probiotic/ton of feed); SIM- (500 g prebiotic + 300 g probiotic/ton of feed).

According to the analysis of the intestinal microbiota, the SIM- treatment resulted in greater richness and uniformity of the microbial community than the other treatments, differing from the CONT+ and PREB treatments in terms of Chao1, observed OTUs, and Fisher’s metrics (Fig 1A–1C), from the SIM+ treatment in terms of Pielou’s evenness metric (Fig 1F), and from the PREB treatment in terms of the Simpson and Shannon metrics (Fig 1D and 1E).

**Fig 2:**
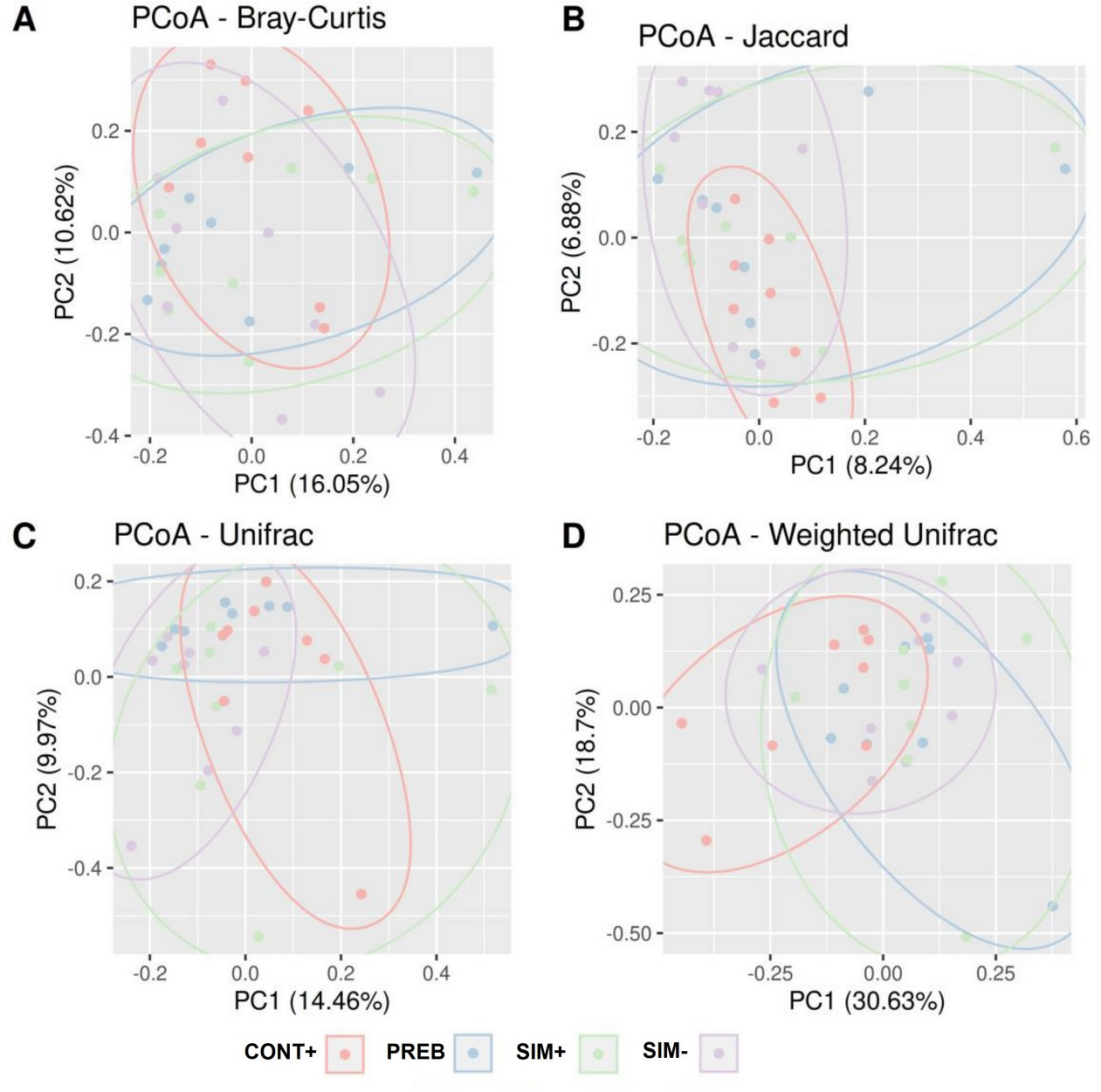
Beta Diversity Estimated by the Bray‒Curtis (A), Jaccard (B), Unweighted UniFrac (C) and Weighted UniFrac (D) Parameters. Colored ellipses were added automatically via the ggforce library in R. CONT+ (200 g halquinol/ton of feed), PREB (1,000 g prebiotic/ton of feed), SIM+ (500 g prebiotic + 600 g probiotic/ton of feed); SIM- (500 g prebiotic + 300 g probiotic/ton of feed).

For beta diversity (Fig 2), a difference in the disparity of taxa present in the CONT+ group was observed, which differed from that in the PREB and SIM+ treatment, as determined on the basis of the abundance and phylogenetic relationships among taxa according to the Bray‒Curtis, Jaccard, and weighted UniFrac parameters (Fig 2A, 2B and 2D). In terms of the phylogenetic relationships among taxa according to the Bray‒Curtis, Jaccard, and weighted UniFrac indices, the PREB, SIM+, and SIM- treatment groups did not differ from each other. There were differences between the CONT+ and SIM- groups according to the Jaccard, unweighted UniFrac, and weighted UniFrac metrics (Fig 2B and 2C). The PREB treatment showed differences compared to the SIM+ and SIM- treatment groups in the UniFrac metric, and the CONT+ group differed from the SIM+ treatment group (Fig 2C).

**Fig 3:**
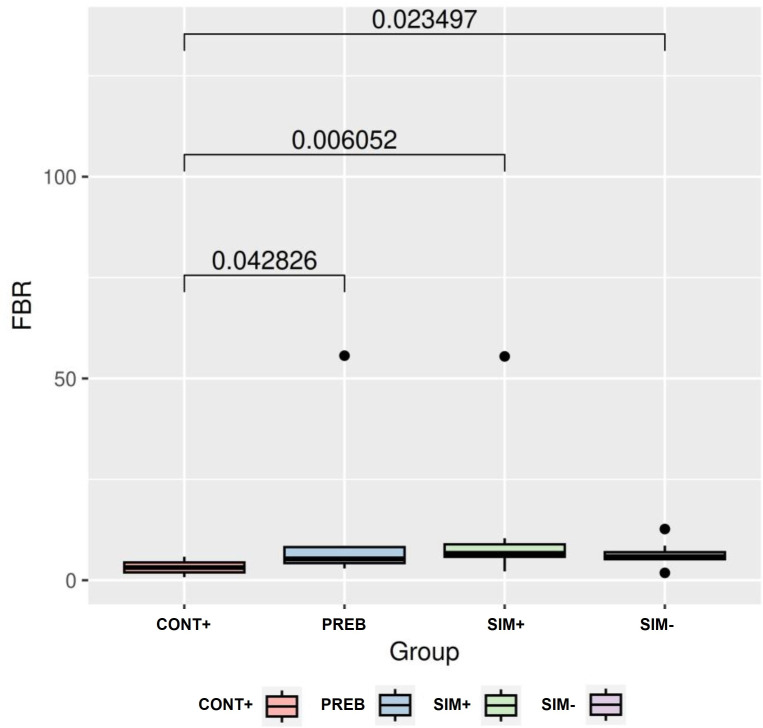
Relationships between *Firmicutes* and *Bacteroidetes* taxa in the groups tested. Differences with p-values less than 0.05 were considered significant. CONT+ (200 g halquinol/ton of feed); PREB (1,000 g prebiotic/ton of feed); SIM+ (500 g prebiotic + 600 g probiotic/ton of feed); SIM- (500 g prebiotic + 300 g probiotic/ton of feed).

Regarding the composition of the bacterial community, the most abundant phyla were *Firmicutes*, *Bacteroidetes*, *Actinobacteria*, and *Proteobacteria*. The *Firmicutes/Bacteroidetes* (F/B) ratio (Fig 3) in the CONT+ group was lower than that in the PREB, SIM+, and SIM- treatment groups.

**Fig 4:**
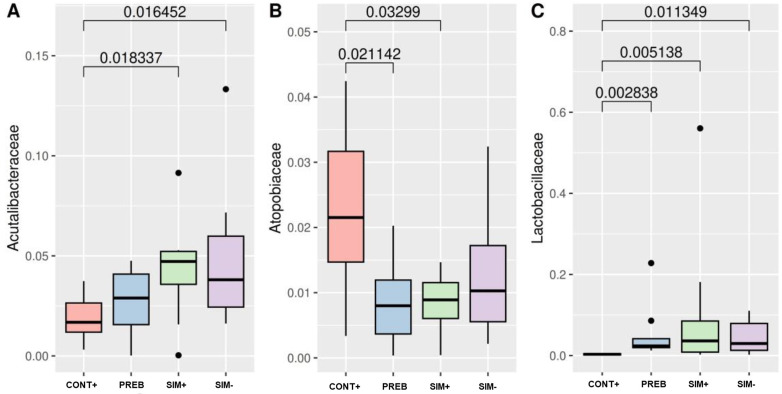
Differential Abundance of the Families *Acutalibacteraceae* (A), *Atopobiaceae* (B) and *Lactobacillaceae* (C). Statistical comparisons between groups were performed using the nonparametric Kruskal‒Wallis test and the Dunn post hoc test. A value less than 0.05 was considered to indicate statistical significance. CONT+ (200 g halquinol/ton of feed); PREB (1,000 g prebiotic/ton of feed); SIM+ (500 g prebiotic + 600 g probiotic/ton of feed); SIM- (500 g prebiotic + 300 g probiotic/ton of feed).

Regarding relative abundance, differences (p<0.05) were observed for the family *Acutalibacteraceae*, with a distinction between the CONT+ group and the SIM+ and SIM- groups (Fig 4A), showing a lower abundance of this family in the PREB group. The family *Atopobiaceae* differed in abundance between the CONT+ group and the PREB and SIM+ groups (Fig 4B), exhibiting a lower abundance in the PREB and SIM+ groups. The abundance of the *Lactobacillaceae* family differed between the CONT+ group and the PREB, SIM+, and SIM- groups (Fig 4C), with the highest abundance in the SIM+ group, followed by the SIM- and PREB groups.

**Fig 5:**
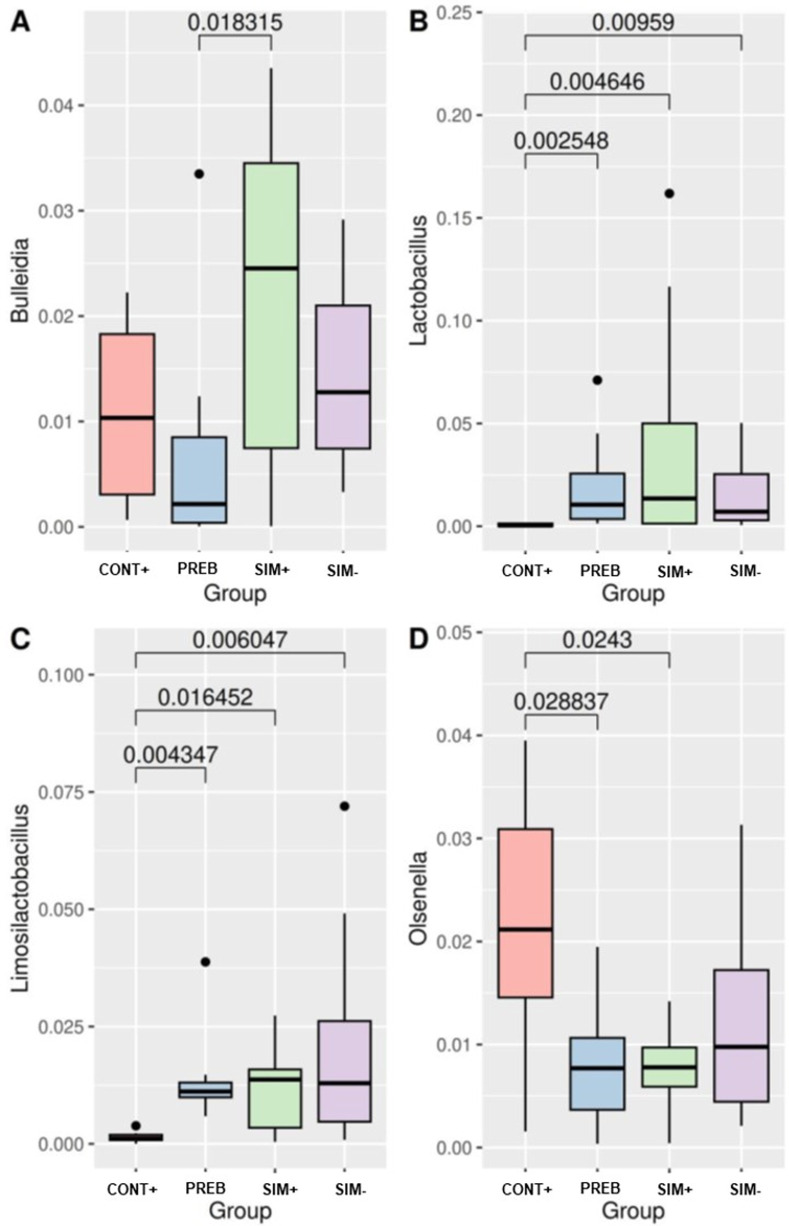
Differential Abundance of the Genera *Bulleidia* (A), *Lactobacillus* (*B)*, *Limosilactobacillus (C) and Olsenella* (D). Statistical comparisons between groups were performed using the nonparametric Kruskal‒Wallis test and the Dunn post hoc test. A value less than 0.05 was considered to indicate statistical significance. CONT+ (200 g halquinol/ton of feed); PREB (1,000 g prebiotic/ton of feed); SIM+ (500 g prebiotic + 600 g probiotic/ton of feed); SIM- (500 g prebiotic + 300 g probiotic/ton of feed).

The abundance of the genera *Bulleidia* differed between the PREB and SIM+ groups (Fig 5A), and the abundance of *Lactobacillus* differed between the CONT+ group and the PREB, SIM+, and SIM- groups (Fig 5B), with a higher abundance in the SIM+ group, followed by the PREB and SIM- groups. The abundance of the genus *Limosilactobacillus* decreased in the CONT+ group compared with that in the PREB and SIM+ treatment groups (Fig 5C); conversely, the abundance of the genus *Olsenella* increased in the CONT+ group compared with that in the PREB and SIM+ treatment groups (Fig 5D).

## Discussion

Piglets receiving the prebiotic (PREB) demonstrated zootechnical performance similar to that of CONT+ group piglets, which is consistent with the findings of Silva et al. [28], who evaluated the performance of weaned piglets fed various prebiotic compositions (including mannooligosaccharides (MOS) and β-glucans, components of the prebiotic used in this study), and Ternus et al. [29], who noted equivalent outcomes in nursery weaning-phase piglets given prebiotics, compared to those treated with colistin, based on the same principles of this study.

MOS, which is not digested in the upper gastrointestinal tract, is fermented by specific bacteria in the colon, leading to the production of regular short-chain fatty acids such as acetate, propionate, and butyrate, along with other metabolites like lactate, pyruvate, ethanol, and succinate, as well as gases such as CO_2_, H_2_, and CH_4_ [7,30,31]. This process minimizes the growth of harmful species such as *Bacteroides*, *Fusobacterium*, and *Clostridium* spp. [32], leading to reduced diarrhea incidence and improved nutrient utilization and performance.

β-glucans enhance plasma leukocyte and lymphocyte proliferation while reducing TNF-α concentrations and fecal *E*. *coli* numbers in weaned piglets [33,34]. These effects may explain why the zootechnical performance of the PREB group was similar to that of the halquinol-treated group (CONT+). Although the final weight gain was similar across all treatments, the poorer FCRs for the SIM+ and SIM- groups than that for the CONT+ group and for the SIM- group than for the PREB group suggest that the lower concentration of prebiotics in the SIM+ and SIM- treatments relative to the dose used in the PREB group, even when combined with a multispecies probiotic, may limit the enhancement of feed conversion efficiency.

Prebiotics, indigestible compounds metabolized by the gut microbiota [35], modulate the nonpathogenic flora [36], suggesting dose-dependent effects. Due to their specific affinity, mannan oligosaccharides (MOSs) can agglutinate pathogens with type 1 fimbriae (e.g., *E*. *coli* and *Salmonella*), preventing their adhesion to the intestinal wall and leading to their excretion [37]. At doses lower than those used in the PREB treatment, the SIM+ and SIM- treatments may have induced less intense pathogen agglutination.

Conversely, the inclusion of a multispecies probiotic in the SIM+ and SIM- treatments might have contributed to the similarity of the weight gain observed across treatment groups by enhancing intestinal epithelium integrity and increasing villus height and crypt depth, thereby expanding the nutrient absorption surface [38], and boosting mucus secretion, which strengthens the intestinal barrier [39]. According to Kodali et al., the presence of *Bacillus coagulans* in animals subjected to SIM+ and SIM- treatments is associated with increased total antioxidant capacity and enzymatic antioxidant activity, as well as the production of extracellular polysaccharides with antioxidant activity [40]. This results in reduced malondialdehyde concentrations in the intestines of weaned piglets and protection against oxidative damage. These properties could account for the similar weight gain and final weight indices observed across all treatments. Similar diarrhea indices between the CONT+ and PREB group and a lower incidence in the SIM- group, particularly compared to that in the CONT+ group, can be attributed to improved intestinal integrity and digestive and absorptive functions following weaning, which are effects mediated by MOS.

Our findings align with those of Silva et al. [41] and Zhao et al. [42], who reported reduced diarrhea incidence in piglets fed MOS-containing diets compared to that in piglets fed diets without MOS. However, the advantages observed, especially for the SIM- group, which had diarrhea scores of 2 and 3, and the similarity of the results for the SIM+ group to the CONT+ group may also be explained by the additional contribution of the probiotic in these treatments.

The synergistic combination of prebiotics and probiotics enhances probiotic stability and survival in the gastrointestinal tract [43]. Prebiotics boost the resilience of probiotic strains by enhancing bacterial adhesion properties and stimulating the activity of preexisting and probiotic species, such as lactobacilli, bacilli, and bifidobacteria. These beneficial microbes compete for adhesion sites on the cellular epithelium, thereby inhibiting pathogenic bacterial colonization [44].

Additionally, this combination promotes intestinal motility, mineral absorption, ammonia removal, and immune system stimulation [45], and Aperce et al. [46] highlighted immunostimulation by *Bacillus licheniformis* and *Bacillus subtilis*. Probiotics also enhance mucus production, reducing the interaction between bacteria and the epithelial barrier [47,48]. The absence of differences in most proinflammatory cytokines at both evaluated ages indicates that the animals were not subjected to a significant infectious challenge. However, at 35 days of age, the level of the cytokine IL-10 increased in the SIM- treatment. IL-10, a key anti-inflammatory mediator linked to prebiotic activity, plays protects the host against pathogens [49].

β-glucans, whose primary receptor Dectin-1 is associated mainly with protein ligands [50], are involved in signaling that induces IL-10 production and respiratory bursts in neutrophils. The lower incidence of diarrhea in the SIM- group correlates with the elevated IL-10 level, which can be attributed to the prebiotic function of additives containing β-glucan.

Prebiotics modulate the immune system by regulating anti-inflammatory cytokine levels [51,52], with some secondary metabolites from their fermentation by probiotic bacteria stimulating the production of IL-10 and other anti-inflammatory markers [53]. This finding indicates that the increase in IL-10 production induced by symbiosis is due to various pathways regulating the expression and production of this anti-inflammatory cytokine. IL-10 can control the duration and intensity of the inflammatory response by inhibiting the production of proinflammatory cytokines such as IL-12p40 [54–57]. The elevated IL-10 levels in the SIM- treatment suggest an anti-inflammatory response to an infectious challenge during the first two weeks of the trial, as evidenced by reduced diarrhea incidence, and a response to a higher concentration of IL-12p40 during this period.

At 49 days of age (28 days post-weaning), the concentration of the cytokine IL-8 was elevated in the SIM- group. This proinflammatory cytokine recruits and activates effector cells of the innate immune response, a phenomenon that can be linked to the function of prebiotics in PREB treatment. IL-8, also known as neutrophil-activating protein-1 (NAP-1), promotes the release of neutrophil granules. Like various chemotactic agents, IL-8 triggers cytoskeletal reorganization, changes in intracellular Ca^2+^ levels, integrin activation, granular protein exocytosis, and respiratory bursts. This activity highlights the value of PREB treatment, indicating the central role of cytokines in immune cell responses and tissue integrity maintenance. Changes in the cytokine network in pig intestines can be expected at weaning [58], aligning with the fact that adaptations were less pronounced in the PREB treatment than in the CONT+ group.

The alpha diversity results (Fig 1) illustrate variations across treatments, with the SIM- group exhibiting the highest diversity. Generally, higher diversity levels support the presence of functionally redundant organisms and greater stability of the intestinal microbiota [59]. Regarding beta diversity evaluation (Fig 2), the Bray‒Curtis, Jaccard, and weighted UniFrac metrics revealed differences between the PREB and SIM+ group compared to the CONT+ group. The modulatory effect of added prebiotics and the symbiotic environment of bacterial populations could explain the observed differences. The SIM- and CONT+ groups also displayed significant differences according to Jaccard and weighted UniFrac indices, reflecting the alpha diversity results found for the SIM- group. In general, specific additives and their combinations have been shown to enhance intestinal microbial diversity in both poultry and mammals [60,61]. In this context, Pan et al. [62] studied the intestinal microbiota of pigs fed diets supplemented with varying doses of xylo-oligosaccharides and found significant differences between the group that received the highest dose of the prebiotic and the negative control group.

Regarding the *Firmicutes/Bacteroidetes* ratio (Fig 3), the CONT+ group had a lower *Firmicutes*/*Bacteroidetes* ratio than did the PREB, SIM+, and SIM- treatment groups, which aligns with the findings of Mulder et al. [63], who, using pigs as a model, reported a strong correlation between the abundance of the *Firmicutes* phylum and a reduction in the incidence of infectious, inflammatory, and autoimmune diseases. This finding explains why the incidence of diarrhea scores 2 and 3 observed for the SIM+ and SIM- treatments did not significantly differ. In this work, it is possible to observe the high rates of relative abundance reported in the different groups, and their increase in treatments PREB, SIM+, and SIM- groups. In addition, the increase in the *Firmicutes/Bacteridota* ratio promoted by diets rich in fiber already were previously reported [64].

The decreased abundance of the *Lactobacillaceae* family, lactic acid-producing bacteria, and *Limosilactobacillus* in the CONT+ group compared to the other treatment groups (Figs 4C, 5B and 5C) underscores their role in intestinal development and integrity. Predominantly found in the postweaning pig intestinal microbiota [65], the abundance of the genus *Lactobacillus* spp., which is more abundant in the SIM+ group, is directly associated with enhanced feed efficiency and weight gain in pigs. This relationship highlights the function of these bacteria in supporting pig health and growth, as evidenced by the work of Le Sciellour et al. [65], Gardiner et al. [66], and Trevisi et al. [67], underscoring the impact of microbial populations on animal nutrition and health outcomes. The genus *Limosilactobacillus*, characterized by rod-shaped or coccoid bacteria, produces exopolysaccharides from sucrose that support biofilm formation on the nonsecretory epithelium in the upper intestinal tract.

Zhang et al. [68] explored the effects of dietary supplementation with *Limosilactobacillus mucosae* on pigs and noted improvements in immunological functions and intestinal microbiota modulation. Although this study did not pinpoint any *Limosilactobacillus* species as solely responsible for modulation of the abundance of this taxon, *L*. *mucosae* was the most abundantly represented species. This finding aligns with the current understanding of the role of probiotics in enhancing gut health and the immune response.

The *Acutalibacteraceae* family was more abundant in piglets fed diets supplemented with prebiotics (SIM+ and SIM-), yet despite being mentioned in several recent studies [68–70], there is no direct reference regarding its significance to the swine intestinal microbiota. The *Atopobiaceae* family, which was most abundant in the CONT+ group and differed in abundance only in the PREB treatment (Fig 4B), also lacks detailed reports about its role in the swine intestinal microbiota. However, these findings are in line with those of Duarte and Kim [71], who noted a decrease in the relative abundance of this family in the gastrointestinal tract of weaning-phase pigs receiving probiotic-supplemented feed. This finding highlights the nuanced and evolving understanding of microbial interactions within the swine gut, underscoring the need for further research into the roles of specific microbial families.

The modulation of the abundance of this family is directly linked to the relative abundance of the *Olsenella* genus, which significantly decreased in both the PREB and SIM+ treatment groups. However, utilizing a combination of probiotics, Oh et al. [72] reported the opposite effect, observing an increase in the relative abundance of the genera *Olsenella*, *Catonella*, *Catenibacterium*, and *Acidaminococcus*. This discrepancy underscores the complex interactions within the gut microbiota, in which the impact of probiotics can vary markedly depending on the specific microbial community and the dietary context. Finally, the SIM+ treatment (Fig 5A) resulted in a greater relative abundance of the genus *Bulleidia*, which belongs to the *Firmicutes* phylum and whose abundance is positively correlated with the concentration of volatile fatty acids [73,74]. This characteristic may be leveraged when utilizing symbiotic compound-based additives, highlighting the potential of symbiotics to enhance beneficial microbial profiles and their metabolic outputs in the gut.

## Conclusions

The use of prebiotics, whether solely or in combination with probiotics, can serve as a viable alternative to AGPs. Our findings suggest that higher doses of prebiotics, either used independently or with probiotics, effectively improve performance parameters, yielding results comparable to those achieved with AGPs. This approach also promotes better modulation of the intestinal microbial community, enhancing gut health and animal growth. For industrial application, we recommend considering the inclusion of prebiotics such as yeast cell wall components in combination with multispecies probiotics in feed formulations. This combination has shown potential not only for improving growth performance but also for supporting the overall health of piglets during the nursery phase, offering a sustainable and health-conscious alternative for the swine industry.
